# The Effectiveness of Probiotics in Psoriasis: An Umbrella Review

**DOI:** 10.1155/jnme/1120062

**Published:** 2026-04-17

**Authors:** Maulidah Ayuningtyas, Farapti Farapti, Siti Sarah Hajar

**Affiliations:** ^1^ Magister of Public Health, Faculty of Public Health, Universitas Airlangga, Surabaya, Indonesia, unair.ac.id; ^2^ Department of Nutrition, Faculty of Public Health, Universitas Airlangga, Surabaya, Indonesia, unair.ac.id; ^3^ Division of Pediatric Emergency and Intensive Care, Faculty of Medicine Universitas Indonesia-Dr. Cipto Mangunkusumo National General Hospital (FKUI-RSCM), Jakarta, Indonesia

**Keywords:** inflammation, probiotics, psoriasis, quality of life, umbrella review

## Abstract

**Background:**

Psoriasis is an immune‐mediated inflammatory skin condition that is chronic and causes a great deal of disease burden, especially for those affected in their most productive years. Gut microbiota, immune parameters and, in turn, psoriasis symptom improvement have recently been associated with the use of probiotics in several different studies.

**Objectives:**

This study aims to conduct an umbrella review of systematic reviews and meta‐analyses of the effectiveness of probiotic supplementation as a possible adjuvant in the treatment of psoriasis.

**Methods:**

This umbrella review is listed in the PROSPERO database (registration number CRD420251130518) and was referenced according to the PRISMA 2020 guidelines. The data were obtained after a systematic search of the literature in Cochrane, Scopus and PubMed. The quality of the methodology was evaluated using the AMSTAR 2 tool; the overlap was evaluated with the corrected covered area (CCA) method.

**Discussions:**

Five systematic reviews and meta‐analyses were included, covering hundreds of adult psoriasis patients. The probiotics studied consisted of both single‐strain and multistrain formulations, sometimes combined with prebiotics. Probiotics are associated with significantly reduced Psoriasis Area and Severity Index (PASI) scores, increased PASI 75 response rates, lowered inflammatory biomarkers (CRP, TNFα, IL‐6) and improved Dermatology Life Quality Index (DLQI). Greater effectiveness was found with multistrain probiotics, treatment duration of ≥ 12 weeks and studies conducted in Asia. Most studies reported good safety and minimal side effects. However, a high overlap among included reviews was observed (CCA = 38.64%), which should be considered when interpreting the findings and their limitations.

**Conclusions:**

Probiotics, particularly multistrain formulations, show potential as a safe and effective adjuvant therapy for reducing psoriasis severity and improving patient quality of life. Further clinical trials are needed to identify the most effective strains and optimal duration of treatment.

## 1. Introduction

Psoriasis is a chronic inflammatory skin disease caused by the immune system. Psoriasis was characterized by keratinocyte hyperproliferation and persistent erythematous plaques [1]. It approximately happened to 2%–3% of the global population, with the highest prevalence up to 11% in Northern Europe [2]. The Global Burden of Disease 2019 study showed more than 4.6 million new cases of psoriasis annually [3]. Psoriasis can affect individuals at any age, but it is most frequent among individuals aged 50–69 years [4]. Disability‐adjusted life years (DALY) showed about psoriasis affects, quality of life and productivity. Although the overall trends were declining, the burden among those aged 30–39 has increased, which potentially reduces the performance and economic contribution of this productive age group [5]. There are several clinical types of the disease, including plaque psoriasis, guttate psoriasis, pustular psoriasis and erythrodermic type psoriasis [6]. Diagnosis was usually only by physical exam, but in rare cases, a skin biopsy may be needed to confirm the diagnosis [7]. Standardized tools like the Psoriasis Area and Severity Index (PASI) and the Dermatology Life Quality Index (DLQI) are used to measure severity and quality‐of‐life impact. These tools are especially important because most people with psoriasis are young adults in their productive years. The side effects, pain and mental disorder, decreased productivity at work, which showed that the disease affects the economy and society as a whole [8].

The pathogenesis of psoriasis is defined by the dysregulation of regulatory T cells and dendritic cells, and also by the interleukin (IL)‐23 or IL‐17 axis [9]. The treatment depends on the severity of the disease and other complications. Individuals with mild psoriasis often use topical treatments, including corticosteroids and vitamin D analog, to reduce symptoms. Moderate‐to‐severe illness may necessitate systemic therapy, such as methotrexate, cyclosporine or biologics targeting TNFα, IL‐17 or IL‐23 [10]. Serum biomarkers, including peptidase inhibitor 3 (PI3), showed potential for evaluating psoriasis and maintaining the treatments [7]. The probiotics supplementations presented a potential treatment for psoriasis, as confirmed by a significant increase in PASI and DLQI scores. The immunomodulatory characteristics of probiotics are strongly related to these clinical changes. For example, probiotics reduce proinflammatory cytokines, such TNF‐α and IL‐6, while raising levels of the anti‐inflammatory cytokine, like IL‐10 [11]. Probiotics are essential for maintaining gut function due to their production of effector molecules like bacteriocins, peptidoglycan and short‐chain fatty acids (SCFAs) that improve immune function, reinforce the intestinal barrier and balance the gut microbiota. These chemicals also affect the neurological system and help stop metabolic illnesses like diabetes and obesity. Probiotics, therefore, function as therapeutic agents that promote gastrointestinal and immunological homeostasis [12]. At a molecular level, probiotics help restore the balance of gut microbiota and encourage the formation of SCFAs, such as acetate and propionate. These acids are very important for lowering inflammation by changing the IL‐23 or Th‐17 pathway [13].

Many primary studies and systematic reviews have stated the role of probiotics in the treatment of psoriasis. The need to conduct an umbrella review to identify, evaluate and synthesize the evidence is warranted, as there are numerous systematic reviews that assess the probiotics and psoriasis symptom relief. This umbrella review will support evidence‐based practice, fill knowledge gaps and provide evidence‐based practice recommendations for future studies regarding the use of probiotics and psoriasis, as well as the various proposed pathophysiological mechanisms. This study is an umbrella review of systematic reviews and meta‐analyses regarding probiotics as adjunct therapy for psoriasis and will examine the types of probiotics, pathophysiological mechanisms, severity of the disease and quality of life. The primary review of the literature regarding the use of probiotics in the treatment of psoriasis has produced contradicting reviews, and this is the reason for conducting an umbrella review to refine the evidence.

## 2. Methods

An umbrella review provides a critical assessment of multiple reviews or meta‐analyses of a specific research question. The most recent developments in systematic evidence synthesis propose a typology for the identification and assessment of core components of evidence. Within such a typology, evidence synthesis aims to evaluatively answer specific questions from evidence users. The synthesis addresses the users’ relational concerns regarding the quantity, dispersion and intricacy of the evidence [14]. Such a typology grants the evidence synthesis community the flexibility to refine the research question while maintaining a close focus, constructive assessment of the answerability and epidemiological breadth of the empirically grounded question [15]. In line with this framework, the present umbrella review was prospectively registered in the International Prospective Register of Systematic Reviews (PROSPERO), ensuring methodological transparency and adherence to a predefined protocol. This typological approach allows the evidence synthesis community to refine research questions while maintaining a focused, critical appraisal of answerability and the epidemiological breadth of empirically grounded questions.

### 2.1. Inclusion Criteria

This review sets boundaries to only systematic reviews and meta‐analyses with study subjects being individuals with a diagnosis of psoriasis. The probiotics of interest were probiotic supplements, both single strain and multistrain, alongside standard psoriasis treatments. The placebo group received supplements and standard treatments. Outcomes included measures of psoriasis severity with PASI, quality of life assessed by the DLQI and any relevant safety problems. Only studies published in English between the years 2000 and 2025 were included.

### 2.2. Study Selection

All of the research team during the study selection carried out an extensive literature search across three major databases: Scopus, PubMed and the Cochrane Library. A systematic set of search terms was developed and documented in an accompanying Excel file, incorporating keywords related to psoriasis, probiotics, PASI, DLQI and productivity. Boolean operators and database‐specific filters were applied to maximize both relevance and timeliness of the retrieved studies. The search was limited to publications between 1 January 2000 and April 2025. All identified references were imported into EndNote for systematic management, where duplicate records were removed, and the remaining studies were screened based on predefined inclusion and exclusion criteria.

## 3. Methodology Report

All the steps of the review process adhered to the PRISMA criteria for study selection and will concentrate on systematic reviews and meta‐analyses found on the internet [16]. The protocol was prospectively registered in the PROSPERO database under the registration number CRD420251130518. The eligible studies will be extracted and presented by all of the research team in a supporting file at Table S2. The primary search terms were *psoriasis, probiotic, psoriasis area severity index, pasi* and *DLQI.* To ensure compliance with international reporting standards, the PRISMA 2020 checklist has been included in the supporting file for this manuscript.

### 3.1. Data Extraction

Two of the authors (MA and F) extracted data from eligible SRMAs, including summary information from the randomized controlled trials (RCTs) reported within them. For every SRMA, the following information was collected: first author’s surname, year of publication, overall participant count, how many primary RCTs the meta‐analysis contained and any additional RCTs found in related reviews. Key features included in the summaries of each SRMA were included in this study design of the RCTs: the number of participants per group and the intervention, sex and mean age of the participants, treatment, type of psoriasis and mean follow‐up duration. Details on probiotic supplementation, including type, administration route, duration, daily dose and probiotic details (genus, species and strain), were also included. Information on additional medication, severity assessment of psoriasis and main outcomes (PASI, DLQI and inflammatory biomarkers: TNFα, IL‐6 and C‐reactive protein [CRP]) was also collected from each SRMA. Table 1 contains the summarized extracted data.

**TABLE 1:** Summary of systematic reviews and meta‐analyses on probiotic supplementation in psoriasis.

Author, Year	PASI score	DLQI score	Other outcomes	Summary	Strength	Limitation
Li et al., 2024	MD = −3.74 (95% CI: −5.83 to −1.66, *p* < 0.001), *I* ^²^ = 73%	SMD = −0.30 (95% CI = −0.92 to 0.32, *p* = 0.34 ⟶ not significant	TNF‐α SMD = −0.83, (95% CI = −1.28–0.37, *p* < 0.001) ⟶ Significant CRP SMD = −1.02, (95% CI = −1.40 to‐0.64, *p* < 0.001) IL‐6 SMD = −0.87 (95% CI = −1.72 to −0.02, *p* < 0.001)	Probiotics significantly reduced disease severity by lowering PASI scores, reducing inflammatory markers and improving quality of life in posttreatment assessments.	Multiple clinically relevant outcomes	No significant association between probiotic use and risk of developing psoriasis High heterogeneity Most studies had short treatment durations Improvements in quality of life by DLQI were only observed in posttreatment

Zeng et al., 2021	SMD = 1.83 (95% CI: 0.41 to −4.07, *p* = 0.11), *I* ^²^ = 96%	N/A	N/A	Some individual trials showed PASI and inflammation improvements; total effective rate better in the probiotic group; low adverse events were reported	First review to comprehensively assess both RCTs and preclinical trials; evaluated multiple outcomes, including efficacy and safety	Only 3 RCTs with small sample sizes; heterogeneity in strains, outcome reporting and participant characteristics; overall low GRADE evidence

Wei et al., 2024	SMD: −1.40 (95% CI: 0.41 to −4.07, *p* < 0.001) *I* ^²^ = 95%	SMD: −0.92 (95% CI: −1.86 0.01, *p* < 0.0001), *I* ^²^ = 50%	↓ TNFα, IL‐6, IL‐17 ↓ Total cholesterol ↓ Triglycerides ↓ Uric acid	Probiotic supplementation significantly reduced PASI and DLQI scores compared to placebo	Comprehensive synthesis of 5 RCTs, PASI and DLQI both assessed, confirms potential of probiotics as adjunctive therapy	Small number of studies; limited sample sizes; clinical scores only (no lab markers); more high‐quality trials needed

Wang et al., 2025	Multistrain probiotics were more effective in improving PASI scores and interventions lasting ≥ 6 months showed greater effects	DLQI improved significantly (NavarroLópez et al., 2019; Zangrilli et al., 2022; Buhaș et al., 2023)	↓ IL‐17a expression (NavarroLópez et al., 2019); ↓ epidermal thickness (Nakamizo et al., 2017)	Probiotic combined with diet improve PASI, DLQI and biomarkers	Clinical and preclinical	High variability in diet protocols, medication confounders, heterogeneity.

Zhu et al., 2024	MD = −3.09 (95% CI: −5.44 to −0.74, *p* = 0.01), I² = 85%	MD = −1.45 (95% CI: −6.72 to 3.82, *p* = 0.59), I² = 85%	CRP: MD = −2.36 (95% CI: −2.77 to −1.95, *p* < 0.001), *I* ^²^ = 0% IL‐6: MD = −1.24 (95% CI: −3.54 to 1.06, *p* = 0.29), *I* ^²^ = 81%	Probiotics significantly improved PASI and CRP levels, but no effect on DLQI or IL6	Included only RCTs; performed subgroup and sensitivity analyses; well‐reported methods	Small sample size; lack of publication bias assessment; heterogeneity in strains and treatment duration

Abbreviations: CRP = C‐reactive protein, IL = interleukin, MD = mean difference; SMD = standardized mean difference, TNF‐α = tumor necrosis factor‐alpha.

### 3.2. Assessment of Methodological Quality

Methodological quality was assessed using the AMSTAR‐2 tool to evaluate the methodological rigour for this review of healthcare interventions on RCTs and non‐RCTs [17]. Analysing PICO questions included applying and assessing different criteria associated with the establishment of study protocols, literature search strategies, the selection of studies, the collection of data and the evaluation of bias in primary research. Seven of the 16 elements are in the primary foundational domains, which enhances the certainty of the outcomes of the review. An element may have the following designations: ‘Yes’, ‘Partial Yes’, ‘No’ or ‘Meta‐analysis not applicable’. A simple algorithm sets a confidence rating: High, Moderate, Low or Critically Low, for both the ‘critical’ and ‘noncritical’ outcomes of that component [18]. In practice, users often face challenges in interpreting and applying the criteria due to ambiguous definitions, variation in judgements among reviewers and lack of explicit guidance for complex cases. Studies have shown that the majority of systematic reviews receive a critically low rating, either due to genuinely poor methodological quality or the stringency of the AMSTAR 2 criteria. Therefore, it is recommended that an appraisal be conducted by a team through consensus and supported with additional decision rules to enhance consistency and transparency. The AMSTAR 2 checklist has been added to the appendix. Therefore, a methodological quality appraisal was reported by two authors (SSH and F), with discrepancies resolved through discussion to achieve consensus. The AMSTAR‐2 checklist is provided in Table 2.

**TABLE 2:** AMSTAR‐2 checklist assessment for methodological quality of included systematic reviews and meta‐analyses.

AMSTAR checklist	SRMA 1	SRMA 2	SRMA 3	SRMA 4	SRMA 5
Did the research questions and inclusion criteria for the review include the components of PICO	Yes	Yes	Yes	Yes	Yes
Protocol established prior to review	Yes	Yes	Yes	Yes	Yes
Study design selection explained	Yes	Yes	Yes	Yes	Yes
Comprehensive literature search	Yes	Yes	Partial yes	Yes	Yes
Study selection in duplicate	Yes	Yes	Yes	No	Yes
Data extraction in duplicate	Yes	Yes	Yes	No	Yes
List of excluded studies with justification	Partial yes	No	No	No	No
Description of included studies	Yes	Yes	Yes	Yes	Yes
Risk of bias assessment	Yes	Partial yes	Yes	Yes	Yes
Reporting of funding in included studies	Yes	No	No	No	No
Meta‐analysis methods	Yes	Yes	Yes	Yes	Yes
Impact of risk of bias on results	Yes	Yes	No	Yes	Yes
Risk of bias considered in interpretation	Yes	Yes	Yes	Yes	Yes
Heterogeneity explanation	Yes	Yes	Yes	Yes	Yes
Publication bias assessment	Yes	Yes	No	No	No
Conflict of interest reported	Yes	Yes	Yes	Yes	Yes
Confidence rating conclusion	Moderate	Low	Critically low	Critically low	Critically low

*Note:* Assessment conducted using the AMSTAR‐2 tool. Categorized rating as Yes, Partial yes or No.

### 3.3. Overlapping Analysis

**FIGURE 1:**
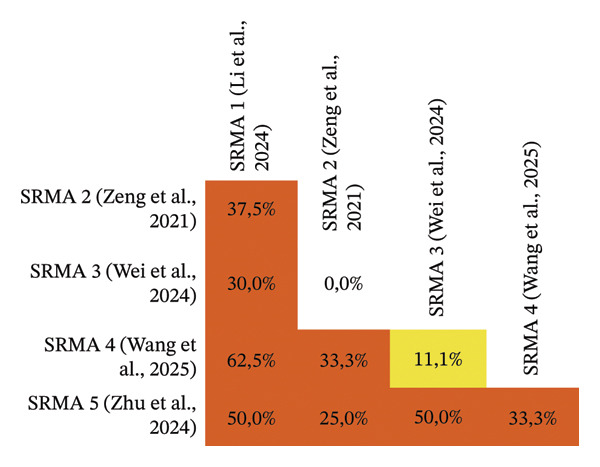
Overlap analysis of primary studies included in the systematic reviews and meta‐analysis.

Overlap analysis by two authors (MA and F) was conducted using the corrected covered area (CCA) method (see Figure 1), which quantifies the level of duplication in the primary studies and helps identify redundancy [19]. For the CCA assessment, the authors employed the Graphical Representation of Overlap for Overviews (GROOVE) tool to evaluate and visualize the overlap of primary studies included in an umbrella review [14].

## 4. Result and Discussion

### 4.1. Study Selection

**FIGURE 2:**
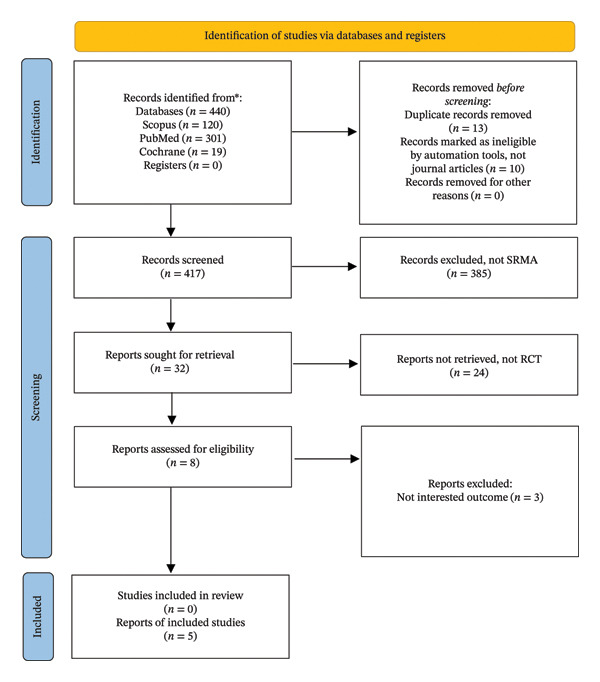
PRISMA 2020 flow diagram of the study selection process ^∗^Consider, if feasible to do so, reporting the number of records identified from each database or register searched (rather than the total number across all databases/registers). ^∗∗^If automation tools were used, indicate how many records were excluded by a human and how many were excluded by automation tools. Source: Page MJ, et al. BMJ 2021; 372:n71. doi: 10.1136/bmj.n71. This work is licenced under CC BY 4.0. To view a copy of this licence, visit https://creativecommons.org/licenses/by/4.0/.

The study selection process is summarized in the PRISMA flow diagram (see Figure 2). A total of 440 records were initially identified through electronic database searching, including PubMed (*n* = 301), Scopus (*n* = 120) and the Cochrane Library (*n* = 19). No records were identified from the trial registers. Prior to screening, 23 records were removed, comprising duplicate records (*n* = 13) and records automatically excluded by screening tools because they were not journal articles (*n* = 10). Following this process, 417 records were screened based on titles and abstracts. Of these, 385 records were excluded because they were not systematic reviews or meta‐analyses. The full texts of 32 reports were subsequently sought for retrieval. However, 24 reports were not retrieved as they did not meet the eligibility criteria, primarily because they were not RCTs. Eight reports were assessed for full‐text eligibility. Of these, three reports were excluded due to outcomes that were not relevant to the objectives of the review. Ultimately, five reports met the inclusion criteria and were included in the final review. Table 1 lists the included studies.

### 4.2. Characteristic Study

A total of five SRMAs were included in this umbrella review and published between 2021 and 2025 [20–24]. These studies compiled primary data from various RCTs involving hundreds of adult patients with psoriasis from different countries (see Supporting Table S1). This reflects a diverse population and geographical context, enhancing the external validity of the findings. The probiotics used in these studies included both single‐strain and multistrain formulations. Single‐strain probiotics used across studies included *Bifidobacterium infantis*, *Streptococcus salivarius* K12, *Lactobacillus rhamnosus* and *Bacteroides fragilis* (BF839). Conversely, four studies employed multiple‐strain probiotic combinations, which included blends such as *Bifidobacterium longum*, *B. lactis* and *Lactobacillus rhamnosus*, as well as *Lactobacillus acidophilus*, *Bifidobacterium bifidum*, *B. lactis* and *B. longum*. Other studies incorporated broader combinations involving *Lactobacillus* spp., *Bifidobacterium* spp., *Enterococcus* spp. and *Bacillus* spp., including strains like *L. brevis*, *L. casei*, *L. gasseri*, *L. reuteri* and *L. plantarum*. A commercial multi strain capsule, Lactocare, was also used in some trials, containing 12 strains of *Lactobacillus*, *Bifidobacteria* and *Streptococcus thermophilus*. In several interventions, these probiotics were paired with prebiotics such as fructo oligosaccharides (FOS), creating synbiotic formulations that aim to enhance both microbial viability and host benefits. One specific combination involved five *Bacillus* strains with added FOS to support gut health and immune function.

The lengths of the interventions across the studies varied from weeks to months. Most reports noted the best outcomes clinically after 12 weeks of use. In the broadest sense, the use of probiotics as primary therapy for psoriasis, due to their safety and noninvasive nature, is something to consider. All five of the SRMAs described the changes in clinical severity in relation to the PASI score. Among the three reviews that described a decrease in PASI scores on the inclusion of probiotics, a lot of variability was found across analyses. One review claimed that the results of the studies combined did not demonstrate noticeable changes, although the studies independently yielded positive outcomes; however, it was attributed to the composition and length of the interventions. The extreme heterogeneity of the studies (*I* ^2^ > 75%) can be attributed to the differing probiotic strains, lengths of interventions and characteristics of the study populations.

Outcomes of quality of life, most often measured with the DLQI, were incorporated in four SRMAs. Statistically significant changes in DLQI scores were noted in two reviews for participants on probiotics versus placebo.

Compared to other SRMAs, there are fewer nonsignificant results and improvements restricted to after‐treatment evaluations. The studies included in the review described the results of the DLQI rather ineptly, which weakens the overall estimates. Of the three SRMAs, two focused on metabolic and inflammatory markers. Findings included the lowered inflammatory markers (TNF‐α, CRP, IL‐6 and IL‐17), altered lipid profile and lowered uric acid. There were, however, inconsistencies among the individual reviews; some reported significant changes, and some reported no significant changes among the markers.

### 4.3. Assessment of Methodological Quality

SRMA 1 by Li et al. [20] was the only study that received a moderate rating in AMSTAR 2, indicating that it had complete documentation and consistent methodological procedures. Almost all critical items, including protocol registration, study selection, risk of bias assessment and meta‐analysis methods, were reported adequately. This makes SRMA 1 a benchmark or reference point compared to the other four SRMAs. Conversely, SRMA 2 was rated low due to the absence of a list of excluded studies (Item 7) and the lack of explicit consideration of how the risk of bias might affect the meta‐analysis results (Item 12). Despite this, technical aspects such as justification of study designs and meta‐analysis methods were well explained. This illustrates that even with solid technical reporting, neglecting transparency in study selection and risk of bias interpretation can significantly lower the overall confidence rating.

SRMA 3, 4 and 5 were all rated critically low, though for different reasons. SRMA 3 appeared the weakest, failing to meet multiple critical items such as funding source reporting, risk of bias assessment and publication bias evaluation. The main weakness of SRMA 4 lay in the lack of duplicate study selection and data extraction, which may compromise the reliability of the data analysed. SRMA 5, while adequately reporting several technical aspects such as PICO, risk of bias and heterogeneity analysis, still received the lowest rating due to the omission of funding information, exclusion list and publication bias assessment. According to the SRMAs, a critically low in AMSTAR 2 rating does not always reflect truly poor quality, but can result from the tool’s strict assessment criteria [18]. AMSTAR 2 ratings are also highly dependent on user interpretation, with variation frequently occurring, especially on ambiguous items. Therefore, it is recommended that appraisals be conducted through team consensus using additional decision rules to improve consistency. Many items such as funding reporting, excluded study lists and publication bias assessment are often not reported because of negligence, but because they are not perceived as mandatory by review authors. However, under AMSTAR 2, these omissions can lead to significant methodological penalties [25]. The AMSTAR 2 Checklist is shown in Table 2.

### 4.4. Degree of Overlap Between SRMA

The analysis conducted using the GROOVE tool indicated that the overall CCA was 38.64% (see Figure 1), corresponding to a very high level of overlap. CCA values below 5% are labelled as *slight* overlap, values from 5% to < 10% as *moderate*, 10% to < 15% as *high* and ≥ 15% as *very high*. The tool’s colour palette is designed to map directly onto these categories, thereby enabling rapid visual appraisal of problematic duplication across reviews. The colour coding applied follows the predefined interpretative thresholds of the GROOVE tool rather than an arbitrary visual choice. In GROOVE, each cell (or ‘node’) represents the CCA calculated for a pairwise comparison between two systematic reviews, and colours are automatically assigned according to categorical cut‐offs that classify the degree of overlap. As described in the methodological guidance, this finding reflects substantial duplication of primary studies across the five included systematic reviews and meta‐analyses [17].

The heatmap illustrates the proportion of overlapping primary studies between pairs of SRMAs on probiotic supplementation in psoriasis, with darker orange shading indicating higher degrees of overlap. The highest overlap was observed between SRMA 1 (Li et al., 2024) and SRMA 4 (Wang et al., 2025), with a shared study overlap of 62.5%, whereas no overlap was identified between SRMA 2 (Zeng et al., [21]) and SRMA 3 (Wei et al., [22]). Overall, the very high CCA value highlights overlap as a key methodological limitation that may affect the robustness and interpretability of findings in umbrella reviews. In light of this substantial overlap, no additional meta‐analysis was undertaken to avoid double‐counting of primary studies and potentially misleading effect estimates. Instead, results are presented descriptively and qualitatively, in accordance with best‐practice recommendations for managing overlap in evidence synthesis.

### 4.5. Impact of Probiotic Supplementation on PASI

Supplementation of probiotics has been shown across almost all of the systematic reviews and meta‐analyses to decrease PASI scores by mean differences (MDs) ranging from 3.09–4.54 points, whereas single‐strain probiotics were shown significantly to be less effective, for example, the study of Li et al. with an MD of −4.61 for multistrain and −2.00 for single strain. Multistrain probiotics have been shown to be more effective for decreasing PASI scores. Some positive subgroup analyses were apparent with prolonged intervention durations, leading to further decreases in PASI scores and increased positive scores in studies conducted in Asia. Probiotics were shown to significantly increase the odds of achieving PASI 75 with the odds of 4.80 times compared to placebo, demonstrating substantial clinical value. Similarly, about lesion clearance, the probiotics increased the likelihood by 3.14 times higher compared to placebo. Those results mean probiotics minimize psoriasis, especially multistrain probiotics, which are more effective than single‐strain probiotics. Multistrain probiotic formulations have been proven to considerably improve the clinical efficacy of managing psoriasis by lowering PASI scores and increasing the odds of obtaining PASI 75 [26].

Probiotics positively affect gut microbiota and inflamed pathways by downregulating TNF‐α and IL‐17, key contributors to the pathogenesis of psoriasis. Probiotics may also improve the strength of epithelial barriers and regulate the immune system via *T*‐regulatory cells [27]. Meanwhile, the use of probiotics from Lactobacillus and Bifidobacterium has been studied extensively, and most of these strains are classified as Generally Recognized As Safe (GRASS) and have very low infection risks to the general population [28]. Probiotics are still considered safe when used in accordance with clinical guidelines and recommended dosages, despite rare instances of opportunistic infections in immunocompromised patients. Probiotics, therefore, are a viable option for psoriasis as a long‐term adjunctive therapy.

### 4.6. Impact of Probiotic Supplementation on DLQI

Findings on the impact of probiotics on the DLQI vary across systematic reviews and meta‐analyses. Several reviews, such as those by Li et al. and Wei et al., reported improvements in DLQI scores following probiotic intervention, with standardized mean differences (SMD) reaching −0.61, particularly in studies involving multistrain probiotics, intervention durations of 12 weeks or longer and Asian populations [29]. However, these findings were not consistent across all studies; for example, Zhu et al. found no statistically significant differences (*p* > 0.05), suggesting that heterogeneity in study design and participant characteristics can influence outcomes [30]. Physiologically, the benefits of probiotics on quality of life are believed to be mediated via the gut skin axis, through which probiotics modulate the gut microbiota, reduce systemic inflammation, enhance mucosal barrier function and regulate immune responses all contributing to both clinical and psychosocial improvements [31]. While DLQI is the most frequently used instrument to assess dermatology‐specific quality of life, research has pointed to several limitations. The original version includes only four response options and often results in irrelevant responses that may distort the total score. To address these issues, revised versions such as DLQI‐Revised or DLQI not scored have been developed by incorporating an additional moderate category and applying statistical adjustments for ‘not relevant’ answers. Recent validation studies have shown that DLQI yields a more sensitive and reliable score distribution, particularly in patients with mild to moderate psoriasis [32]. Moreover, DLQI scores are influenced by sociodemographic factors. Men often have higher PASI scores, while women report worse DLQI scores, especially regarding emotional well‐being, work limitations and sexual life, indicating that social and psychological burdens may disproportionately affect women [33].

### 4.7. Effect of Probiotics on Inflammatory Biomarkers in Psoriasis

In response to the probiotic intervention, a number of clinical studies showed a marked decrease in the activity indicators of inflammation in the integumentary system, such as CRP (SMD = 1.02; *p* < 0.001), TNF‐α (SMD = −0.83; *p* < 0.001) and IL‐6 (−0.87; *p* = 0.04), thus resulting in the interest of some clinical researchers regarding the role of probiotics in decreasing systemic inflammation in psoriasis. Probiotics influence the immune system blockade at the intestinal mucosal layer. If a balance of good microorganisms (probiotics) is maintained, they produce anti‐inflammatory SCFAs (especially butyrate) [34]. The inflammatory cytokines in the bloodstream were decreased, and then, the immune system response in the infected tissue and the inflammatory response system would result in the psoriasis inflammatory response system (in the bloodstream), resulting in decreased SMD values. Furthermore, probiotics are able to increase the number of regulatory T cells (Tregs) and decrease the activity of Th17 and Th1 cells, resulting in a decrease in proinflammatory cytokines such as IL‐6, TNF‐α and IL‐1β [35]. Besides all that, probiotics also improve the intestinal mucosal barrier, lower systemic endotoxemia and reduce the activity of the NFκB pathway (which is very important in triggering the inflammatory response).

While the positive anti‐inflammatory effects from the studies may be valid (if we ignore the absence of negative effects), the considerable heterogeneity among studies (*I* ^2^ > 70%) needs to be considered for the interpretation of the results. The differences in strains of the probiotics used, amounts, the duration of the intervention and the characteristics of the participants (study population) may explain this heterogeneity. The biomarker reductions, especially CRP, and the correlating levels of TNFα and IL6 should be viewed in the context of the systemic and psoriatic disease associated with the comorbidities of cardiovascular disease and metabolic syndrome [34]. Meanwhile, the controversial, low‐cost, safe and noninvasive probiotic intervention could be employed as a possible adjunct treatment for psoriasis, if future systematic studies confirm these initial findings. More of these systematic medical studies should be conducted to assess the duration of the intervention and the strains of the probiotics used in order to determine the effects of the probiotics on the aforementioned parameters.

### 4.8. Safety and Adverse Effects of Probiotics in Psoriasis Patients

Overall, probiotics were considered safe, with a low incidence of adverse effects reported across all included SRMAs, including those by Zeng et al. and Zhu et al. Most studies indicated good tolerability, and no serious adverse events directly attributed to probiotic use were identified. Furthermore, there was no significant association between probiotic supplementation and an increased risk of new‐onset psoriasis. These findings support the general safety profile of probiotics when used as an adjunctive therapy in psoriasis management. However, continued monitoring in larger and longer term studies is recommended to confirm these safety outcomes.

## 5. Conclusions

This umbrella review demonstrates that probiotic supplementation, particularly multistrain formulations and administration for ≥ 12 weeks, has potential as a safe and effective adjuvant therapy in the management of psoriasis. Probiotics have been shown to increase the PASI score, increase PASI 75 achievement, decrease inflammatory biomarkers (CRP, TNF‐α, IL‐6) and improve patients’ quality of life or DLQI. However, the high degree of heterogeneity and substantial overlap across studies represent key methodological limitations. Therefore, further clinical trials with improved design and diverse populations are needed to identify the most effective strains, optimal dosage and ideal treatment duration. These findings support probiotics as a promising, noninvasive approach to psoriasis therapy through microbiota and immune system modulation.

## Author Contributions

Maulidah Ayuningtyas was responsible for conception and design of the study, literature search, data extraction and drafting manuscript. Siti Sarah Hajar was responsible for conception and design of the study, literature search, data extraction and drafting the manuscript. Farapti Farapti provided supervision, critical revision of the manuscript, methodological validation and final approval of the version to be submitted.

## Funding

This research did not receive any specific grant from funding agencies in the public, commercial or not‐for‐profit sectors.

## Conflicts of Interest

The authors declare no conflicts of interest.

## Supporting Information

The materials in supporting file provide additional information supporting this study, an umbrella review of the probiotics in psoriasis. The Supporting Table S1 showed the detailed characteristics of the included primary studies (systematic reviews and meta‐analysis), the guidelines reported, and the last is the GROOVE tool to assess overlap among primary studies.

Table S1. Summary of Objectives, Interventions and Outcomes of Systematic Reviews Assessing Probiotics in Psoriasis.

This table summarizes main characteristics of the included primary studies of the systematic reviews and meta‐analyses in this review. Information was shown to the author, year of publication, number of included RCTs, sample size, probiotic interventions, outcome measures (such as PASI, DLQI and inflammatory biomarkers) and key findings.

Table S2. PRISMA 2020 Checklist.

This table was presented the PRISMA 2020 checklist that reporting the checklist of this umbrella review. Each checklist item was shown where the corresponding information can be found in the main manuscript.

## Supporting information


**Supporting Information** Additional supporting information can be found online in the Supporting Information section.

## Data Availability

The data that support the findings of this study are available in the supporting information of this article.
