# Supplementary material for: The Effectiveness of Probiotics in Psoriasis: An Umbrella Review
Source: J Nutr Metab. 2026 Apr 17;2026:1120062. doi: 10.1155/jnme/1120062 (PMC13089384; doi:10.1155/jnme/1120062)
Supplement: Supplementary file 1 — Supporting Information Additional supporting information can be found online in the Supporting Information section. [file JNME-2026-1120062-s001.docx]

**Supplementary Materials**

**Table S1. Summary of Objectives, Interventions, and Outcomes of Systematic Reviews Assessing Probiotics in Psoriasis**

| No | Author  Year | Objective of the Review | Inclusion Criteria | Number of Primary Studies | Type of Intervention | Outcomes | Population Characteristics |
| --- | --- | --- | --- | --- | --- | --- | --- |
| 1 | Li., 2024 | Impact of probiotic supplementation on psoriasis severity, including changes in PASI score, PASI 75 response, clearance rates, life quality outcomes, and inflammatory markers. | P (Population):  Adults with psoriasis (from clinical trials and NHANES datasets)  I (Intervention):  Probiotic supplementation (various strains, doses, and durations)  C (Comparison):  Placebo or no probiotic intervention  O (Outcomes):  Primary: Reduction in PASI score  Achievement of PASI 75  Clearance rate  Improvement in quality of life (DLQI or similar)  Secondary:  Decrease in inflammatory biomarkers (CRP, IL-6, TNF-α | 8 RCT and 3 Non RCT | Single-strain probiotics:  Bifidobacterium infantis  Streptococcus salivarius K12  Lactobacillus rhamnosus  Bacteroides fragilis (BF839)  Mixed/multi-strain probiotics:  Kombinasi dari:  Lactobacillus acidophilus  Bifidobacterium bifidum  Bifidobacterium longum  Enterococcus spp.  Bacillus spp.  Lactobacillus brevis, casei, gasseri, reuteri, plantarum | Primary Outcomes   - Differences in PASI scores between the probiotic and placebo groups. - Changes in PASI scores from baseline to post-probiotic intervention. - Achievement of PASI 75 response. - Lesion clearance rates, including complete resolution of skin lesions. - Improvements in quality of life (QoL) following probiotic intervention. - Changes in QoL scores from baseline to post-intervention.   Secondary Outcomes   - Changes in C-reactive protein (CRP) levels. - Changes in interleukin-6 (IL-6) concentrations. - Changes in tumour necrosis factor-alpha (TNF-α) levels. | Age  Median = 60 years (IQR: 45–72)  Sex  Male: 322 (42.1%)  Female: 442 (57.9%)  Education Level  Less than high school: 135 (17.7%)  High school graduate: 436 (57.1%)  Above high school: 193 (25.2%)  Poverty-Income Ratio (PIR)  Low: 251 (32.9%)  Medium: 256 (33.5%)  High: 257 (33.6%)  Body Mass Index (BMI)  Underweight/normal weight: 192 (25.1%)  Overweight: 250 (32.7%)  Obese: 322 (42.1%)  Physical Activity Level  Never: 286 (37.4%)  Low: 116 (15.2%)  Intermediate: 121 (15.8%)  High: 120 (15.7%)  Very high: 121 (15.8%) |
| 2 | Zeng et al.,  2021 | To evaluate the effectiveness and safety of probiotic supplementation in the treatment of psoriasis through systematic review and meta-analysis of RCTs and preclinical trials. | Participants (P):  Adult patients diagnosed with psoriasis by a physician with adequate clinical qualifications.  Diagnosis based on established clinical criteria for psoriasis.  No restriction on whether it is a first-time diagnosis or a recurrence.  Intervention (I):  The intervention group received probiotic supplements, with no restriction on bacterial species, content, etc.  Comparison (C):  The control group received a placebo or other non-probiotic interventions.  Outcomes (O):  Assessed outcomes included:  PASI (Psoriasis Area and Severity Index)  Inflammatory indicators  Total effective rate  Adverse events  Study Design:  Only Randomized Controlled Trials (RCTs)  Exclusion Criteria:  Non-RCT studies  Non-adult patients | 3 RCTs (164 participants) | Single-strain probiotic  Bifidobacterium infantis 35624  Multiple-strain probiotic  Bifidobacterium longum, B. lactis, Lactobacillus rhamnosus  Combination of Bifidobacterium, Lactobacillus, Enterococcus, Bacillus | PASI  Secondary Outcomes  CRP  TNF-α  IL-6 | Population Characteristics (Human Studies – RCTs)  Total number of participants: 164 individuals across 3 RCTs  Countries involved: Spain, Ireland, China  Age range: Adults aged 18–70 years  Gender: Not consistently reported  Psoriasis type: Primarily plaque psoriasis  Severity: Mild to moderate (e.g., PASI > 6 or PASI < 16) |
| 3. | Wei et al.,  2025 | To systematically evaluate the efficacy of probiotic supplementation in the treatment of psoriasis. | Population (P):  Patients diagnosed with psoriasis  Intervention (I):  Probiotic supplementation  Comparison (C):  Placebo  Outcome (O):  Improvement in PASI (Psoriasis Area and Severity Index)  Improvement in DLQI (Dermatology Life Quality Index) | 5 studies (RCTs), total 286 patients | Single-Strain Probiotics  Lactobacillus rhamnosus  Multi-Strain Probiotics  Lactobacillus acidophilus  Bifidobacterium bifidum  Bifidobacterium lactis  Bifidobacterium longum  Lactobacillus acidophilus  Bifidobacterium bifidum  Bifidobacterium lactis  Bifidobacterium longum  Synbiotic formulation:  Lactobacillus spp.  Bifidobacterium spp.  Streptococcus thermophilus  Fructo-oligosaccharides (FOS) | Primary Outcomes  PASI (Psoriasis Area and Severity Index)  DLQI (Dermatology Life Quality Index):  Secondary Outcomes (from discussion, not pooled in meta-analysis)  ↓ Inflammatory cytokines (e.g., TNF-α, IL-6, IL-17)  ↓ Cholesterol  ↓ Triglycerides  ↓ Uric acid | Total sample size: 286 participants  Intervention group: 143  Control group: 143  Age: median age around 18+ years,all ≥18 years  Diagnosis: clinically diagnosed with psoriasis  Gender: balanced male-female ratio  Disease type:  Most participants had plaque-type psoriasis  Setting:  Studies were conducted in clinical or hospital-based settings |
| 4 | Wang et al.,  2025 | To evaluate and summarize evidence on the effects of different dietary patterns and components on psoriasis, aiming to provide evidence-based dietary recommendations. | P – Population:  Patients with psoriasis (various types and severity levels), including those with plaque psoriasis, vulgaris psoriasis, and psoriatic arthritis.  I – Intervention:  Administration of probiotics or a combination of probiotics and prebiotics as a dietary intervention.  C – Comparison:  Control groups received placebo (such as maltodextrin) or standard topical treatment only.  O – Outcome:  Changes in PASI (Psoriasis Area and Severity Index), DLQI (Dermatology Life Quality Index), and inflammatory markers such as CRP, IL-6, and TNF-α. | 42 clinical studies and 11 preclinical studies | Single-Strain Probiotics  Bifidobacterium infantis 35264  Streptococcus salivarius K12  Lactobacillus rhamnosus  Multiple-Strain (Mixed) Probiotics  Kombinasi: B. longum + B. lactis + L. rhamnosus  Kombinasi 5 strain Bacillus sp. + prebiotik (FOS, | PASI  DLQI  CRP  IL-6  TNF alfa | Type of Psoriasis:  Plaque psoriasis: 4 out of 5 studies → 80%  Vulgaris psoriasis: 1 out of 5 studies → 20%  Disease Severity:  Mild to moderate: 3 out of 5 studies → 60%  Active: 1 out of 5 studies → 20%  Not specified: 1 out of 5 studies → 20%  Country of Study:  Europe (Spain, Ireland, Italy, Romania): 4 out of 5 → 80%  South America (Brazil): 1 out of 5 → 20% |
| 5. | Zhu et al.,  2025 | To evaluate the efficacy and safety of probiotic supplementation in the treatment of psoriasis | P (Population/Patient):  Adults (≥18 years old) diagnosed with psoriasis, based on PASI or other clinical criteria.  I (Intervention):  Administration of oral probiotics as an adjuvant therapy (various strains, including Bifidobacterium, Lactobacillus).  C (Comparison):  Placebo or control without probiotics.  O (Outcomes):  Primary: Changes in PASI (Psoriasis Area and Severity Index), DLQI (Dermatology Life Quality Index), and levels of inflammatory markers (e.g., CRP, IL-6).  Secondary: Incidence of adverse events (AEs). | 7 RCT | 4 studies used multiple strains of probiotics, including combinations of:  Bifidobacterium longum, B. lactis, Lactobacillus rhamnosus  Lactobacillus acidophilus, B. bifidum, B. lactis, B. longum  A commercial capsule (Lactocare®) with 12 strains including Lactobacillus, Bifidobacteria, and Streptococcus thermophilus, with prebiotic (FOS)  3 studies used single strain of probiotics:  Bifidobacterium infantis 35,264  Lactobacillus rhamnosus (in two separate studies, capsule and drink form) | PASI (Psoriasis Area and Severity Index)  PASI 75  DLQI (Dermatology Life Quality Index)  CRP (C-Reactive Protein)  IL-6 (Interleukin-6) | Age 18-70 yo  Gender : male and female  The majority of studies included plaque psoriasis, particularly mild to moderate forms  Average Duration of Probiotic Therapy 8 to 12 weeks |

**Table S2. PRISMA 2020 Checklist**

| **Section and Topic** | **Item #** | **Checklist item** | **Location where item is reported** |
| --- | --- | --- | --- |
| **TITLE** | | |  |
| Title | 1 | Identify the report as a systematic review. | The Effectiveness of Probiotic in Psoriasis: An Umbrella Review |
| **ABSTRACT** | | |  |
| Abstract | 2 | See the PRISMA 2020 for Abstracts checklist. | Abstract page (includes background, methods, results, conclusion) |
| **INTRODUCTION** | | |  |
| Rationale | 3 | Describe the rationale for the review in the context of existing knowledge. | Introduction,burden of psoriasis & role of probiotics explained |
| Objectives | 4 | Provide an explicit statement of the objective(s) or question(s) the review addresses. | Introduction, explicit aim: to assess probiotic effectiveness in psoriasis |
| **METHODS** | | |  |
| Eligibility criteria | 5 | Specify the inclusion and exclusion criteria for the review and how studies were grouped for the syntheses. | Eligibility criteria section – inclusion of Meta Analysisi, psoriasis patients, probiotic intervention, PASI/DLQI outcomes |
| Information sources | 6 | Specify all databases, registers, websites, organisations, reference lists and other sources searched or consulted to identify studies. Specify the date when each source was last searched or consulted. | Study selection section – PubMed, Scopus, Cochrane mentioned (but no search date → missing) |
| Search strategy | 7 | Present the full search strategies for all databases, registers and websites, including any filters and limits used. | No detailed search string provided → missing |
| Selection process | 8 | Specify the methods used to decide whether a study met the inclusion criteria of the review, including how many reviewers screened each record and each report retrieved, whether they worked independently, and if applicable, details of automation tools used in the process. | Study selection section – selection process described, but only **single reviewer** (PRISMA requires ≥2 independent reviewers) |
| Data collection process | 9 | Specify the methods used to collect data from reports, including how many reviewers collected data from each report, whether they worked independently, any processes for obtaining or confirming data from study investigators, and if applicable, details of automation tools used in the process. | Data extraction *section* – extraction form described, no double-checking mentioned |
| Data items | 10a | List and define all outcomes for which data were sought. Specify whether all results that were compatible with each outcome domain in each study were sought (e.g. for all measures, time points, analyses), and if not, the methods used to decide which results to collect. | PASI and DLQI |
|  | 10b | List and define all other variables for which data were sought (e.g. participant and intervention characteristics, funding sources). Describe any assumptions made about any missing or unclear information. | Probiotic strain, duration, geographic region, SRMA quality |
| Study risk of bias assessment | 11 | Specify the methods used to assess risk of bias in the included studies, including details of the tool(s) used, how many reviewers assessed each study and whether they worked independently, and if applicable, details of automation tools used in the process. | Methodological quality – assessed with **AMSTAR-2** |
| Effect measures | 12 | Specify for each outcome the effect measure(s) (e.g. risk ratio, mean difference) used in the synthesis or presentation of results. | Reported as Mean Difference (MD) with 95% CI |
| Synthesis methods | 13a | Describe the processes used to decide which studies were eligible for each synthesis (e.g. tabulating the study intervention characteristics and comparing against the planned groups for each synthesis (item #5)). | Subgroup analyses (strain, duration, region), heterogeneity (I²), CCA overlap. No sensitivity analysis reported. |
|  | 13b | Describe any methods required to prepare the data for presentation or synthesis, such as handling of missing summary statistics, or data conversions. | Subgroup analyses (strain, duration, region), heterogeneity (I²), CCA overlap. No sensitivity analysis reported. |
|  | 13c | Describe any methods used to tabulate or visually display results of individual studies and syntheses. | Subgroup analyses (strain, duration, region), heterogeneity (I²), CCA overlap. No sensitivity analysis reported. |
|  | 13d | Describe any methods used to synthesize results and provide a rationale for the choice(s). If meta-analysis was performed, describe the model(s), method(s) to identify the presence and extent of statistical heterogeneity, and software package(s) used. | Subgroup analyses (strain, duration, region), heterogeneity (I²), CCA overlap. No sensitivity analysis reported. |
|  | 13e | Describe any methods used to explore possible causes of heterogeneity among study results (e.g. subgroup analysis, meta-regression). | Subgroup analyses (strain, duration, region), heterogeneity (I²), CCA overlap. No sensitivity analysis reported. |
|  | 13f | Describe any sensitivity analyses conducted to assess robustness of the synthesized results. | Subgroup analyses (strain, duration, region), heterogeneity (I²), CCA overlap. No sensitivity analysis reported. |
| Reporting bias assessment | 14 | Describe any methods used to assess risk of bias due to missing results in a synthesis (arising from reporting biases). | Not described → missing |
| Certainty assessment | 15 | Describe any methods used to assess certainty (or confidence) in the body of evidence for an outcome. | No GRADE or certainty of evidence reported → missing |
| **RESULTS** | | |  |
| Study selection | 16a | Describe the results of the search and selection process, from the number of records identified in the search to the number of studies included in the review, ideally using a flow diagram. | PRISMA flowchart included; number of SRMAs reported |
|  | 16b | Cite studies that might appear to meet the inclusion criteria, but which were excluded, and explain why they were excluded. | No table of excluded studies with reasons → missing |
| Study characteristics | 17 | Cite each included study and present its characteristics. | Study characteristics table included |
| Risk of bias in studies | 18 | Present assessments of risk of bias for each included study. | AMSTAR 2 results described narratively, but not shown in a detailed table |
| Results of individual studies | 19 | For all outcomes, present, for each study: (a) summary statistics for each group (where appropriate) and (b) an effect estimate and its precision (e.g. confidence/credible interval), ideally using structured tables or plots. | PASI and DLQI results reported (tables/summary) |
| Results of syntheses | 20a | For each synthesis, briefly summarise the characteristics and risk of bias among contributing studies. | Subgroup analyses presented (strain, duration, region); high heterogeneity noted |
|  | 20b | Present results of all statistical syntheses conducted. If meta-analysis was done, present for each the summary estimate and its precision (e.g. confidence/credible interval) and measures of statistical heterogeneity. If comparing groups, describe the direction of the effect. | Subgroup analyses presented (strain, duration, region); high heterogeneity noted |
|  | 20c | Present results of all investigations of possible causes of heterogeneity among study results. | Subgroup analyses presented (strain, duration, region); high heterogeneity noted |
|  | 20d | Present results of all sensitivity analyses conducted to assess the robustness of the synthesized results. | Subgroup analyses presented (strain, duration, region); high heterogeneity noted |
| Reporting biases | 21 | Present assessments of risk of bias due to missing results (arising from reporting biases) for each synthesis assessed. | Not addressed |
| Certainty of evidence | 22 | Present assessments of certainty (or confidence) in the body of evidence for each outcome assessed. | Not reported |
| **DISCUSSION** | | |  |
| Discussion | 23a | Provide a general interpretation of the results in the context of other evidence. | Results interpreted in context of psoriasis therapy |
|  | 23b | Discuss any limitations of the evidence included in the review. | Not explicitly stated |
|  | 23c | Discuss any limitations of the review processes used. | Not described (e.g., single reviewer, limited search) |
|  | 23d | Discuss implications of the results for practice, policy, and future research. | Included – clinical implications for psoriasis therapy |
| **OTHER INFORMATION** | | |  |
| Registration and protocol | 24a | Provide registration information for the review, including register name and registration number, or state that the review was not registered. | Registered process in PROSPERO (ID: 1130518) |
|  | 24b | Indicate where the review protocol can be accessed, or state that a protocol was not prepared. | No PROSPERO link provided |
|  | 24c | Describe and explain any amendments to information provided at registration or in the protocol. | Not reported |
| Support | 25 | Describe sources of financial or non-financial support for the review, and the role of the funders or sponsors in the review. | No funding reported |
| Competing interests | 26 | Declare any competing interests of review authors. | No conflict of interest statement |
| Availability of data, code and other materials | 27 | Report which of the following are publicly available and where they can be found: template data collection forms; data extracted from included studies; data used for all analyses; analytic code; any other materials used in the review. | No data availability statement |

| \| **Overall results** \| \| \| \| --- \| --- \| --- \| \|  \|  \|  \| \| Number of columns (number of reviews) \| c \| 5 \| \| Number of rows (number of index publications) \| r \| 11 \| \| Number of included primary studies (including double counting) \| N \| 28 \| \| Covered area \| N/(rc) \| 50,91% \| \| Corrected covered area \| (N-r)/(rc-r) \| 38,64% \| \| Interpretation of overlap \| **Very High overlap** \| \| \| Structural Zeros \| X \| 27 \| \| Corrected covered area  (adjusting by structural zeros) \| (N-r)/(rc-r-X) \| 100,00% \| \|  \|  \|  \| \| N° of non-overlapped primary studies \| In 1 SR \| 2 \| \| Number of overlapped primary studies \| In 2 SRs \| 4 \| \| In 3 SRs \| 2 \| \| In 4 SRs \| 3 \| \| In 5 SRs \| 0 \| \| In 6 SRs \| 0 \| \| In 7 SRs \| 0 \| \| In 8 SRs \| 0 \| \| In 9 SRs \| 0 \| \| In 10 SRs \| 0 \| \| In 11 SRs \| 0 \| \| In 12 SRs \| 0 \| \| In 13 SRs \| 0 \| \| In 14 SRs \| 0 \| \| In 15 or more SRs \| 0 \| | \|  \| SRMA 1(Li et al., 2024) \| SRMA 2(Zeng et al., 2021) \| SRMA 3(Wei et al., 2024) \| SRMA 4 (Wang et al., 2025) \| \| --- \| --- \| --- \| --- \| --- \| \| SRMA 2(Zeng et al.,  2021) \| 37,5% \| \| SRMA 3(Wei et al.,  2024) \| 30,0% \| 0,0% \| \| SRMA 4 (Wang et  al., 2025) \| 62,5% \| 33,3% \| 11,1% \| \| SRMA 5(Zhu et al.,  2024) \| 50,0% \| 25,0% \| 50,0% \| 33,3% \| |
| --- | --- | --- | --- | --- | --- | --- | --- | --- | --- | --- | --- | --- | --- | --- | --- | --- | --- | --- | --- | --- | --- | --- | --- | --- | --- | --- | --- | --- | --- | --- | --- | --- | --- | --- | --- | --- | --- | --- | --- | --- | --- | --- | --- | --- | --- | --- | --- | --- | --- | --- | --- | --- | --- | --- | --- | --- | --- | --- | --- | --- | --- | --- | --- | --- | --- | --- | --- | --- | --- | --- | --- | --- | --- | --- | --- | --- | --- | --- | --- | --- | --- | --- | --- | --- | --- |

Figure S1. Assessment the CCA of Primary Study Overlap Using GROOVE Tool

| Table S2. Search strategy and boolean operators used for database queries |
| --- |
| ALL ( psoriasis ) |
| TITLE-ABS-KEY ( psoriasis ) |
| ( ALL ( psoriasis ) ) OR ( TITLE-ABS-KEY ( psoriasis ) ) |
| ALL ( probiotic ) |
| TITLE-ABS-KEY ( probiotic ) |
| ( ALL ( probiotic ) ) OR ( TITLE-ABS-KEY ( probiotic ) ) |
| ALL ( "psoriasis area severity index" ) |
| TITLE-ABS-KEY ( "psoriasis area severity index" ) |
| ( ALL ( "psoriasis area severity index" ) ) OR ( TITLE-ABS-KEY ( "psoriasis area severity index" ) ) |
| ALL ( pasi ) |
| TITLE-ABS-KEY ( pasi ) |
| ( TITLE-ABS-KEY ( pasi ) ) OR ( ALL ( pasi ) ) |
| ( ALL ( "psoriasis area severity index" ) ) OR ( ALL ( pasi ) ) |
| ALL ( "dermatology life quality index" ) |
| TITLE-ABS-KEY ( "dermatology life quality index" ) |
| ( ALL ( "dermatology life quality index" ) ) OR ( TITLE-ABS-KEY ( "dermatology life quality index" ) )( ALL ( "dermatology life quality index" ) ) OR ( TITLE-ABS-KEY ( "dermatology life quality index" ) ) |
| ALL ( "psoriasis area severity index" ) ) OR ( ALL ( pasi ) ) OR ( ALL ( "dermatology life quality index" ) ) |
| ALL ( psoriasis ) ) AND ( ALL ( probiotic ) ) AND ( ( ALL ( "psoriasis area severity index" ) ) OR ( ALL ( pasi ) ) OR ( ALL ( "dermatology life quality index" ) ) ) |
